# Chlamydia Induces Anchorage Independence in 3T3 Cells and Detrimental Cytological Defects in an Infection Model

**DOI:** 10.1371/journal.pone.0054022

**Published:** 2013-01-07

**Authors:** Andrea E. Knowlton, Larry J. Fowler, Rahul K. Patel, Shannon M. Wallet, Scott S. Grieshaber

**Affiliations:** 1 Department of Oral Biology, College of Dentistry, University of Florida, Gainesville, Florida, United States of America; 2 Department of Periodontology Biology, College of Dentistry, University of Florida, Gainesville, Florida, United States of America; 3 Department of Pathology, College of Medicine, University of Florida, Gainesville, Florida, United States of America; University of California Merced, United States of America

## Abstract

*Chlamydia* are Gram negative, obligate intracellular bacterial organisms with different species causing a multitude of infections in both humans and animals. *Chlamydia trachomatis* is the causative agent of the sexually transmitted infection (STI) Chlamydia, the most commonly acquired bacterial STI in the United States. Chlamydial infections have also been epidemiologically linked to cervical cancer in women co-infected with the human papillomavirus (HPV). We have previously shown chlamydial infection results in centrosome amplification and multipolar spindle formation leading to chromosomal instability. Many studies indicate that centrosome abnormalities, spindle defects, and chromosome segregation errors can lead to cell transformation. We hypothesize that the presence of these defects within infected dividing cells identifies a possible mechanism for *Chlamydia* as a cofactor in cervical cancer formation. Here we demonstrate that infection with *Chlamydia trachomatis* is able to transform 3T3 cells in soft agar resulting in anchorage independence and increased colony formation. Additionally, we show for the first time *Chlamydia* infects actively replicating cells *in vivo*. Infection of mice with *Chlamydia* results in significantly increased cell proliferation within the cervix, and in evidence of cervical dysplasia. Confocal examination of these infected tissues also revealed elements of chlamydial induced chromosome instability. These results contribute to a growing body of data implicating a role for *Chlamydia* in cervical cancer development and suggest a possible molecular mechanism for this effect.

## Introduction

*Chlamydiae* are bacterial pathogens that infect epithelial cells and are responsible for a wide range of diseases in both animal and human hosts. *Chlamydia trachomatis*, a human pathogen, is comprised of over 15 distinct serovars some of which are responsible for trachoma; the leading cause of preventable blindness, as well as the most commonly acquired sexually transmitted infection of bacterial origin. In women, untreated genital infections can result in devastating consequences such as pelvic inflammatory disease, ectopic pregnancy, and even infertility [1], [2]. Every year, there are over 4 million new cases of Chlamydia in the United States [3], [4] and an estimated 92 million cases worldwide [5]. *Chlamydia muridarum*, formerly the murine serovar of *C. trachomatis* (MoPn), is a natural respiratory pathogen of mice and is used extensively as a model for studying human reproductive tract disease. Infection of mice with *C. muridarum* closely resembles the pathology of genital infection with *C. trachomatis* [6]–[8]. Despite the differences in species tropism *C. trachomatis* and *C. muridarum* share a very similar genome [9]–[11].

Infection with *Chlamydia trachomatis* has been epidemiologically linked to increased rates in cervical cancer in women who are co-infected with human papillomavirus (HPV) [12]–[19]. Cervical cancer is the second most common cancer of women worldwide [20] Greater than 90% of cervical cancers are associated with high risk HPV types 16 and 18, but there is a considerable time gap between exposure to HPV and development of cervical cancer [21]. This is attributed to the fact that HPV is a necessary but insufficient cause of cervical cancer, and many studies have been conducted to investigate other risk factors that are involved in progression of the disease including smoking, exposure to hormones, the host immune system, and presence of other STIs [19], [22].

Chlamydial infection of cells in culture causes significant cytological changes. These changes include centrosome amplification, inhibition of centrosome clustering, and premature mitotic exit. These effects lead to chromosome instability as demonstrated by increased micronuclei formation and increased formation of multinucleated cells [23]–[25]. These cellular defects are apparent in cancerous lesions of almost every origin [26]–[31]. We hypothesize that these transformative defects induced by chlamydial infection can contribute to cellular transformation *in vitro* and *in vivo*.

In this study we show host cell division and not co-expression of any particular viral oncogene is the critical requirement for chlamydial induced cell defects to arise. We demonstrate chlamydial infection can transform 3T3 cells *in vitro* leading to anchorage independence and the formation of colonies in soft agar. Additionally, we utilize the *C. muridarum* mouse model of chlamydial genital tract infection to demonstrate infection of actively replicating cells in the cervical epithelium. We also determine infection with *Chlamydia* induces significant increases in cell proliferation within the cervix in mice and this was consistent in mice that were transgenic for HPV oncoprotein E7 as well as their wild-type littermates. The induction of cytological defects leading to chromosome instability in actively replicating cells is likely an important factor in defining a role for *Chlamydia* in cervical cancer development.

## Results

### The Chlamydial Induced Cytopathic Effects of Centrosome Amplification, Multipolar Spindles, and Multinucleation are Dependent on Cellular Replication and Not Dependent on Coexpression of the E6 and E7 Oncogenes

**Figure 1:**
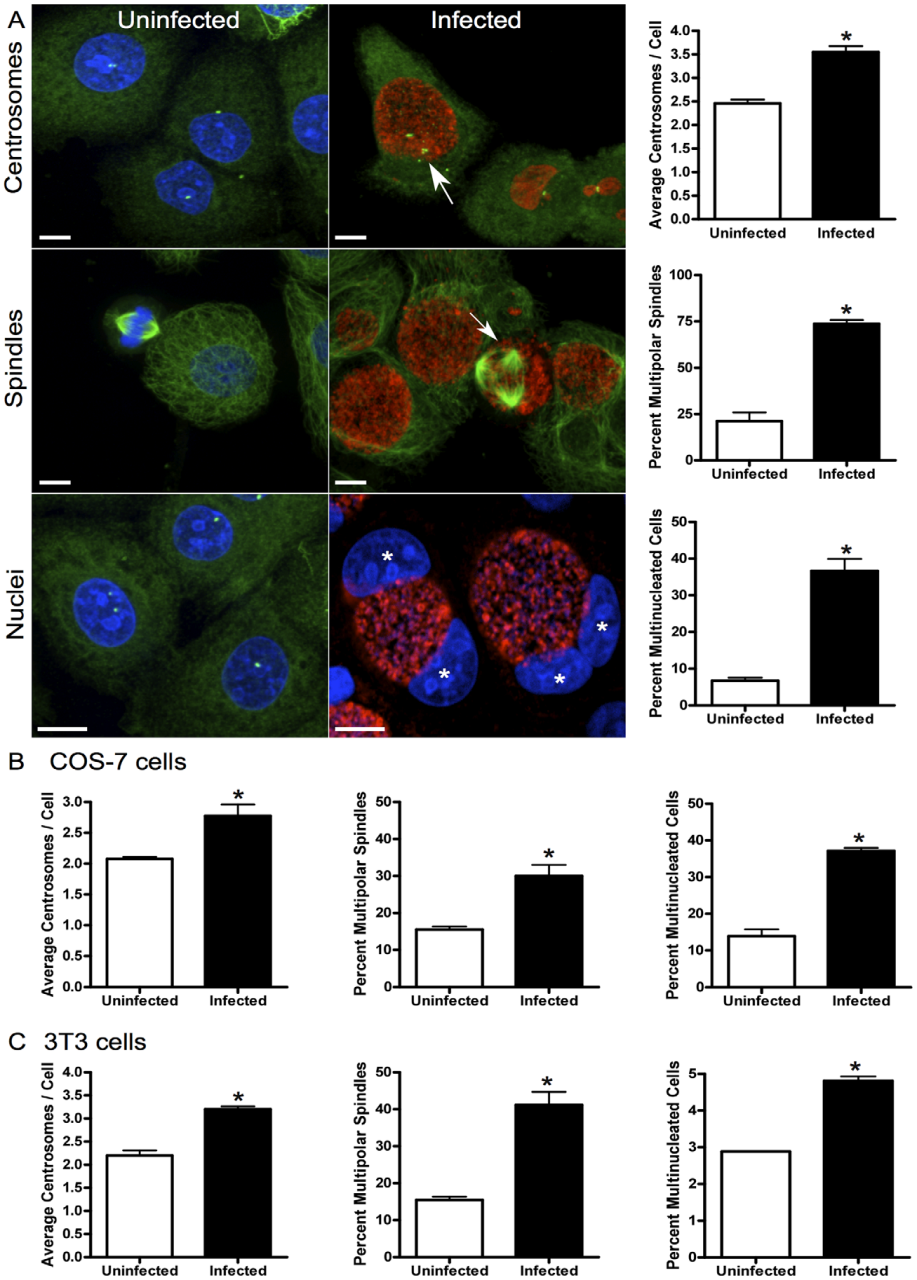
*Chlamydia* induces centrosome and spindle defects in replicating cells. (A) End1/E6E7 cells were stained for centrosomes (green, top and bottom panel), mitotic spindles (green, middle panel), DNA (blue), and *Chlamydia* (red, infected panel). Uninfected cells had an average of 2.5±0.1 centrosomes/cell, and infected cells increased to an average of 3.5±0.1 centrosomes/cell, p<0.0001, N>150 (arrow). Uninfected End1s have an average of 21.2±4.6 percent multipolar spindles (arrow), and the infected cells to an average of 73.7±2.0 percent, p = 0.0005, N>150. The presence of multinucleated cells (stars) increased with infection from 6.7±0.8 to 36.6±3.3 percent, p = 0.0009, N>200. (B) COS-7 cells were treated and stained as above. Uninfected cells had 2.1±0.03 centrosomes/cell, and infection resulted in an increase to 2.8±0.2 centrosomes/cell, p = 0.0200, N>300. Uninfected COS-7s had 15.5±0.8 percent multipolar spindle formation, infected cells increased to 30.0±2.9 percent multipolar spindles, p = 0.0093, N>150. Uninfected cells were 13.9±1.9 percent multinucleated, and infected cells increased to 37.1±0.8 multinucleated, p = 0.0003, N>200. (C) Uninfected and infected 3T3 fibroblasts were evaluated as above. Uninfected 3T3 cells 2.2±0.1 centrosomes/cell, infected cells increased to 3.2±0.1 centrosomes/cell, p = 0.0014, N>300. Uninfected cells had 15.5±0.9 percent multipolar spindles, infected cells increased to 41.2±3.5 multipolar, p = 0.0021, N>150. Uninfected 3T3s were 2.9±0.01 percent multinucleated, and upon infection became 4.8±0.1 percent multinucleated, p<0.0001, N>200. Scale bars, 5 μm.

We have previously described that chlamydial infection induces centrosome amplification, multipolar spindles, and early anaphase onset leading to multinucleation in HeLa cells [23]–[25] HeLa cells are a cervical cancer cell line that express components of the HPV18 genome including the E6 and E7 oncoproteins [32]. Expression of these oncogenes is strongly linked with centrosome amplification and multinucleation [33]. We and others have demonstrated that the induction of multipolar spindles, centrosome amplification, and multinucleation caused by chlamydial infection require progression through the cell cycle [24], [34], [35]. To determine if the oncogenes expressed in HeLa cells were required for any of these phenotypes we measured the rates of centrosome amplification, multipolar spindle formation, and multinucleation in a variety of cells that replicate in culture, including End1/E6E7, COS-7, and 3T3 cells (Figure 1). End1 (ATCC CRL-2615) cells are an endocervical cell line established from normal epithelial tissue and immortalized by transduction with the retroviral vector LXSN-16E6E7 [36]. These cells express the E6 and E7 oncogenes from HPV-16. COS-7 cells are an African green monkey kidney fibroblast-like cell line derived by transformation with an origin defective mutant of SV40 which codes for wild-type T antigen [37]. The 3T3 cell line was established from disaggregated Swiss mouse embryos and spontaneously developed immortality but retain anchorage dependence [38].

We infected End1, COS-7, and 3T3 cells for 36 hours with *C. trachomatis*, serovar L2. We chose this time point because it corresponds with the appearance of centrosome and spindle abnormalities as we have previously described. Compared with their uninfected counterparts, infected End1 cells had elevated numbers of centrosomes, from 2.5±0.1 to 3.5±0.1 centrosomes/cell, respectively. These infected cells also displayed a significant increase in multipolar spindle formation, from 21.2±4.6 percent in uninfected cells to 73.7±2.0 percent. Upon infection End1 cells also demonstrated a significant increase in the percentage of multinucleated cells from 6.7±0.8 to 36.6±3.3 percent (Figure 1A). We also investigated the effects of infection on COS-7 cells, and like the End1 cells, infection led to increased numbers of centrosomes, from 2.1±0.03 centrosomes/cell to 2.8±0.2, respectively. There was also a significant increase in the formation of multipolar spindles after chlamydial infection from 15.5±0.8 percent multipolar to 30.0±2.9 percent. Multinucleated cells also accumulated significantly from 13.9±1.9 percent multinucleated to 37.1±0.8 percent following infection (Figure 1B). Infection of 3T3 fibroblasts displayed a similar trend, with a significant increase in centrosome numbers from 2.2±0.1 to 3.2±0.1 centrosomes/cell. The formation of multipolar spindles upon infection also increased significantly from 5.5±0.9 percent multipolar to 41.2±3.5 percent. The presence of multinucleated 3T3s increased moderately from 2.9±0.01 percent to 4.8±0.1 percent multinucleated following infection (Figure 1C). Although infected 3T3 cells had only a modest but significant increase in multinucleated cells, infection with *Chlamydia* was still able to induce centrosome abnormalities and spindle assembly difficulties. In all cases after infection cells contained amplified centrosomes, increased rates of multipolar spindles and an accumulation of multinucleated cells.

These results support the hypothesis that expression of HPV E6 and E7 or any other specific oncogene are not required for the phenotypic effects induced by chlamydial infection. When considered with published data showing these phenotypes are dependent on the cell cycle [24], [34], [35]; the data suggest that the only cellular cofactors required for these effects is cellular replication.

### Infection of NIH3T3 Cells Induces Anchorage Independence

Centrosome amplification and genomic instability results in permanent changes to the cell. To determine if these potentially transforming phenotypes induced by chlamydial infection could lead to cellular transformation we infected 3T3 cells with *C. trachomatis* L2 at a MOI of 10 to reach a high probability that every cell was infected. These cells were cured of the infection with rifampicin for four days and allowed to recover for an additional three days. The cured cell population produced no infectious progeny as measured by a replating assay; there was no evidence of *Chlamydia* when these cells were stained with pooled human serum (reacts to chlamydial LPS) and no evidence of chlamydial DNA when co-stained with the nucleic acid stain DRAQ5 ( Figure S1).

**Figure 2:**
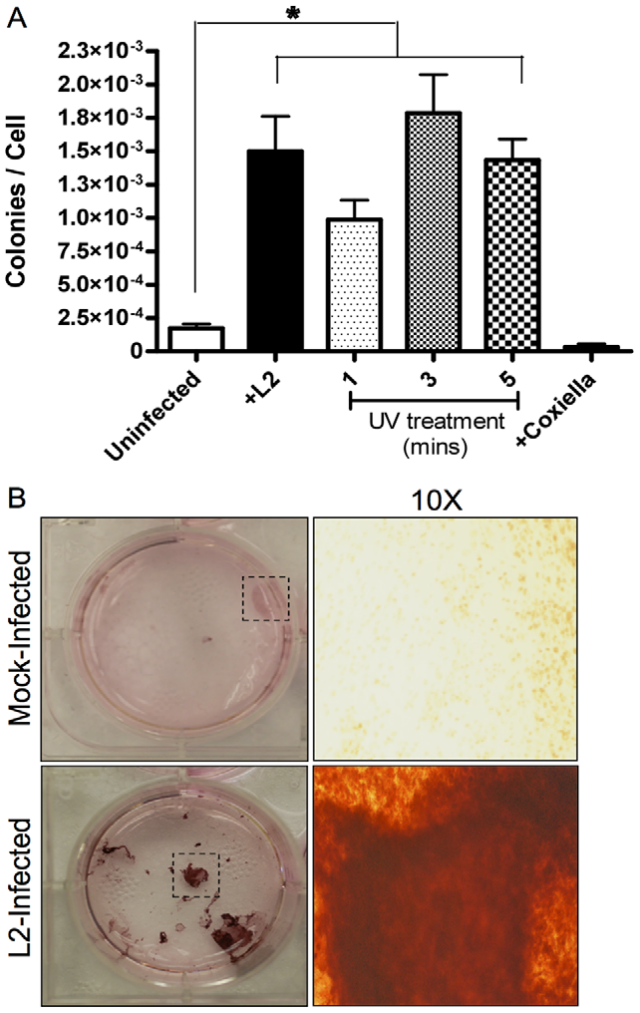
Chlamydial infection induces anchorage independence in 3T3 fibroblasts. Mock-infected and infected 3T3s were cured of chlamydial infection and incubated for 4 weeks in soft agar. The cells were stained, and the colonies were enumerated and normalized to the 2500 cells initially plated. (A) The uninfected cells had an average of 1.7×10^−4^ ±3.1×10^−5^ colonies/cells plated, the infected cells an average of 1.5×10^−3^ ±2.6×10^−4^ colonies/cells plated. Cells treated with UV light for 1 minute had an average of 9.9×10^−4^ ±1.5×10^−4^ colonies/cells plated, while cells treated for 3 and 5 minutes had an average of 1.8×10^−3^ ±2.9×10^−4^ and 1.4×10^−3^ ±1.6×10^−4^ colonies/cells plated, respectively. 3T3 cells cured of an infection with the intracellular bacterial pathogen *Coxiella burnetii* had an average of 3.3×10^−5^ ±2.3×10^−5^ colonies/cells plated. N>72 wells, p<0.0001. (B) The images in the panels are examples of stained colonies from mock-infected and L2-infected cells after a 4 week incubation. The first column is a single well of a 6-well plate. The second column is a 10X magnification of the indicated area (dashed box).

These cured 3T3 cells were plated in soft agar and allowed to grow for 28 days (Figure 2B). *In vitro* cellular transformation detection assays are commonly used to measure the morphological changes in cellular phenotypes induced by carcinogens and other insults [39]–[41]. The 3T3 anchorage-independence assay is an effective tool used to evaluate the mutagenic potential of a chemical or cellular insult [42]. Transformation associated with phenotypic changes, such as 3T3 anchorage-independent growth, can be easily assayed by quantifying colony formation in soft agar [43]. When the infected and cured 3T3s were plated in soft agar we saw a significant increase in colony formation compared to mock-infected and cured 3T3s; the mock-infected cells had an average transformation rate of 1.7×10^−4^ ±3.1×10^−5^ colonies/cells plated while the cured 3T3s had a dramatic increase in colony formation with an average of 1.5×10^−3^ ±2.6×10^−4^ colonies/cells plated (Figure 2A). As a control for transformation we exposed 3T3 cells to ultraviolet (UV) light for 1, 3, and 5 minutes. We saw a significant increase in colony formation at 1 and 3 minutes compared to mock-infected colonies, 9.9×10^−4^ ±1.5×10^−4^ colonies/cells plated and 1.8×10^−3^ ±2.9×10^−4^, respectively. At 5 minutes of UV exposure there was a decrease in the number of colonies/cells plated compared to 1 and 3 minutes, at 1.4×10^−3^ ±1.6×10^−4^, which we attribute to cell death due to excessive DNA damage. To verify that colony formation from infected and cured cells was a *Chlamydia*-specific effect and not the result of intracellular infection or of secondary effects of a large bolus of material like the inclusion, we also infected cells with the obligate intracellular bacterium *Coxiella burnetii. C. burnetii* lives within a parasitophorous vacuole (PV) inside the host cell, and the volume of the *Coxiella* PV can occupy a large portion of the cytoplasm much like the chlamydial inclusion [44]. When the cells infected and then cured of *C. burnetii* were plated in soft agar there was no increase in colony formation compared to mock-infected controls at 3.3×10^−5^ ±2.3×10^−5^ colonies/cells plated. These data indicate that *C. trachomatis* has the ability to induce permanent changes in previously infected cells; changes that can lead to transformation. These observations strengthen the hypothesis that cellular defects that arise due to chlamydial infection have potentially long term detrimental effects.

### Reproductive Tract Infection of Mice Demonstrates *Chlamydia* Infects Replicating Cell Populations

**Figure 3:**
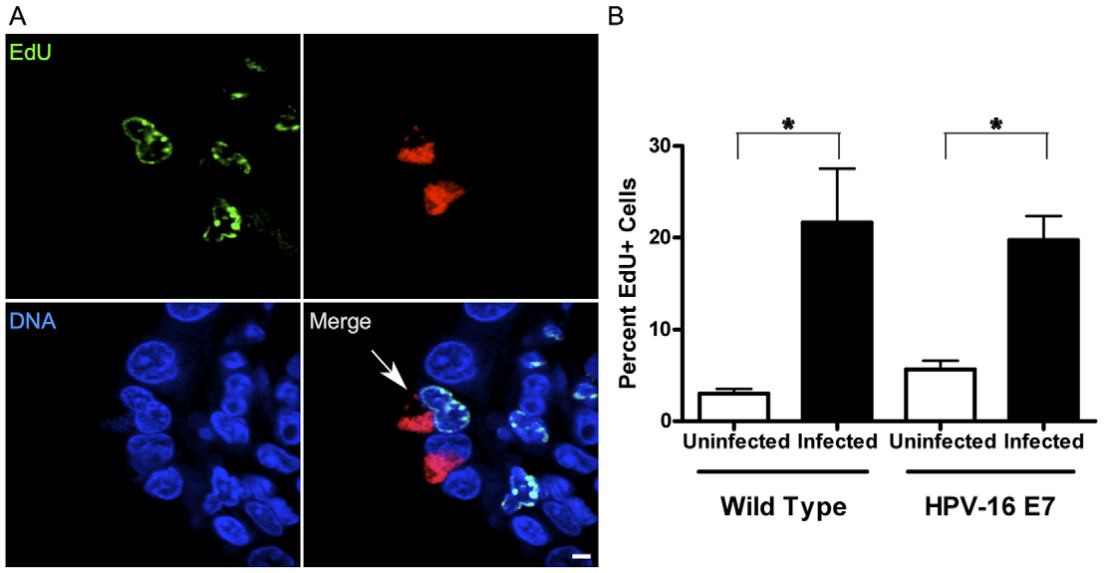
*Chlamydia* infects actively replicating cells *in vivo*, and induces cell proliferation. (A) A female FVB wild-type mouse infected with *Chlamydia muridarum* for 7 days, was treated with EdU (green) prior to sacrifice to detect cell proliferation, and formalin-fixed, paraffin-embedded sections (4 μm thick) were stained for *Chlamydia* (red) and DNA (blue). The sections were imaged by confocal microscopy. A chlamydial inclusion can be seen associated with an actively replicating EdU positive cell (arrow) within the cervical epithelium. (B) Female K14-HPV-E7 mice and their wild-type littermates were mock-infected and infected with *Chlamydia muridarum* for 7 days. The mice were treated with EdU, and the EdU positive cells per total cells present in multiple fields of view were counted to determine the rate of cell proliferation. Wild-type uninfected mice, N = 4, had an average of 3.0±0.5 percent cell proliferation, while the infected WT mice, N = 3, experienced a significant increase of cell proliferation at an average of 21.7±5.9 percent, p = 0.003. The uninfected E7 mice had an average of 5.7±1.0 percent cell proliferation, and the infected E7 mice had a significantly higher average of 19.7±2.6 percent, p = 0.0002, N = 4 mice/group. Scale bars, 5 μm.

*Chlamydia trachomatis* causes sexually transmitted disease infecting the epithelial cells of the vagina, cervix, uterus, and Fallopian tubes. This population of cells is terminally differentiated but does undergo cyclical cell turnover and replacement; consequently there is a constant subset of cells undergoing cellular replication [45]. We used the mouse model of chlamydial infection to ascertain *Chlamydia's* ability to infect this indigenous replicating cell population. We infected 8 week old wild-type FVB/N mice with *Chlamydia muridarum* for 7 days, and injected them with EdU (5-ethynyl-2′-deoxyuridine), a thymidine analog, for three consecutive days prior to sacrifice. The EdU allowed us to visualize any cells that had undergone S-phase, as the thymidine analog would have been taken up by any newly synthesized DNA [46]. EdU incorporation was visualized by reacting tissue sections with a fluorescent azide using click chemistry [46]. We examined cervical epithelial tissue sections co-stained for *Chlamydia*, DNA, and EdU fluorescence. Throughout the vaginal epithelium we observed numerous instances of actively replicating cells infected with *Chlamydia* suggesting that infection of replicating cells *in vivo* happens with a regular frequency (Figure 3A).

### Infection Stimulates Cellular Replication During Infection

Chlamydial infection begins in the vaginal epithelium, ascending toward the cervix and the upper genital tract. The epithelial lining of the vagina and outer cervix is composed of squamous epithelium. Where the outer cervix meets the inner cervix the squamous epithelium is replaced by glandular columnar epithelium, and the columnar epithelium lines the inner cervix and the rest of the upper genital tract. The junction on the cervix where squamous epithelium transitions to columnar epithelium is known as the transformation zone. Because of the high degree of metaplasia most cervical cancers originate in the transformation zone [47], [48].

We have established that the induction of cytological changes during chlamydial infection requires a replicating cell population in culture. Additionally, we hypothesize that the increased cellular replication caused by HPV infection can provide a greater number of replicating target cells creating an environment conducive to these chlamydial induced changes.

We wanted to compare the native replication rate of the cells in the transformation zone with replication rates in the HPV-16 E7 gene knock-in mouse. These mice were created as a model for HPV-induced cervical cancer in an effort to elucidate the E7 oncogene specific contributions to cancer progression. The expression of the E7 oncogene is driven under the human keratin 14 (K14) promoter, restricting its expression to the stratified epithelium. These mice are designated K14-HPV-E7 [49]. The expression of the transgene leads to increased epithelial cellular replication rates over the wild-type animals. These animals were also treated with exogenous estrogen, as it has been shown to be an essential cofactor in the onset and development of cervical cancer in this mouse model [50].

To determine the proliferation rates of the cervical epithelium in these animals we calculated the percentage of EdU positive cells present over multiple fields of view within the transformation zone (Figure 3B). The wild-type, mock-infected animals had a native cell proliferation rate of 3.0±0.5 percent while the K14-HPV-E7 mice had a native replication rate of 5.7±1.0 percent.

We also looked at the effect infection had on replication rates in the transformation zone. All infected animals had a considerably higher proliferation rate of 19.7±2.6 percent and 21.7±5.9 percent for the wild type and K14-HPV-E7, respectively. We believe the increase in cell proliferation rates due to chlamydial infection can be attributed to remodeling of the epithelial lining after the insult of infection. However, upon reinfection this remodeling gives *Chlamydia* overwhelming access to actively replicating cells, resulting in the opportunity to induce transformative defects within the host cell. The bacterial load for each infected animal was calculated based on recovered inclusion forming units (IFUs) and was similar for all mice (Table 1).

**Table 1:** Inclusion forming units (IFU) recovered from animals on day 1 postinfection.

Wild-type	IFU	K14-HPV-E7	IFU
(N = 3)	1.28×10_7_	(N = 4)	8.42×10_5_
	2.48×10^7^		1.52×10_6_
	5.07×10^5^		3.56×10_6_
			1.11×10_7_

Summary of live bacterial shedding from wild-type and K14-HPV-E7 infected mice.

### Chlamydial Infection Induces Cervical Dysplasia in Mice

**Figure 4:**
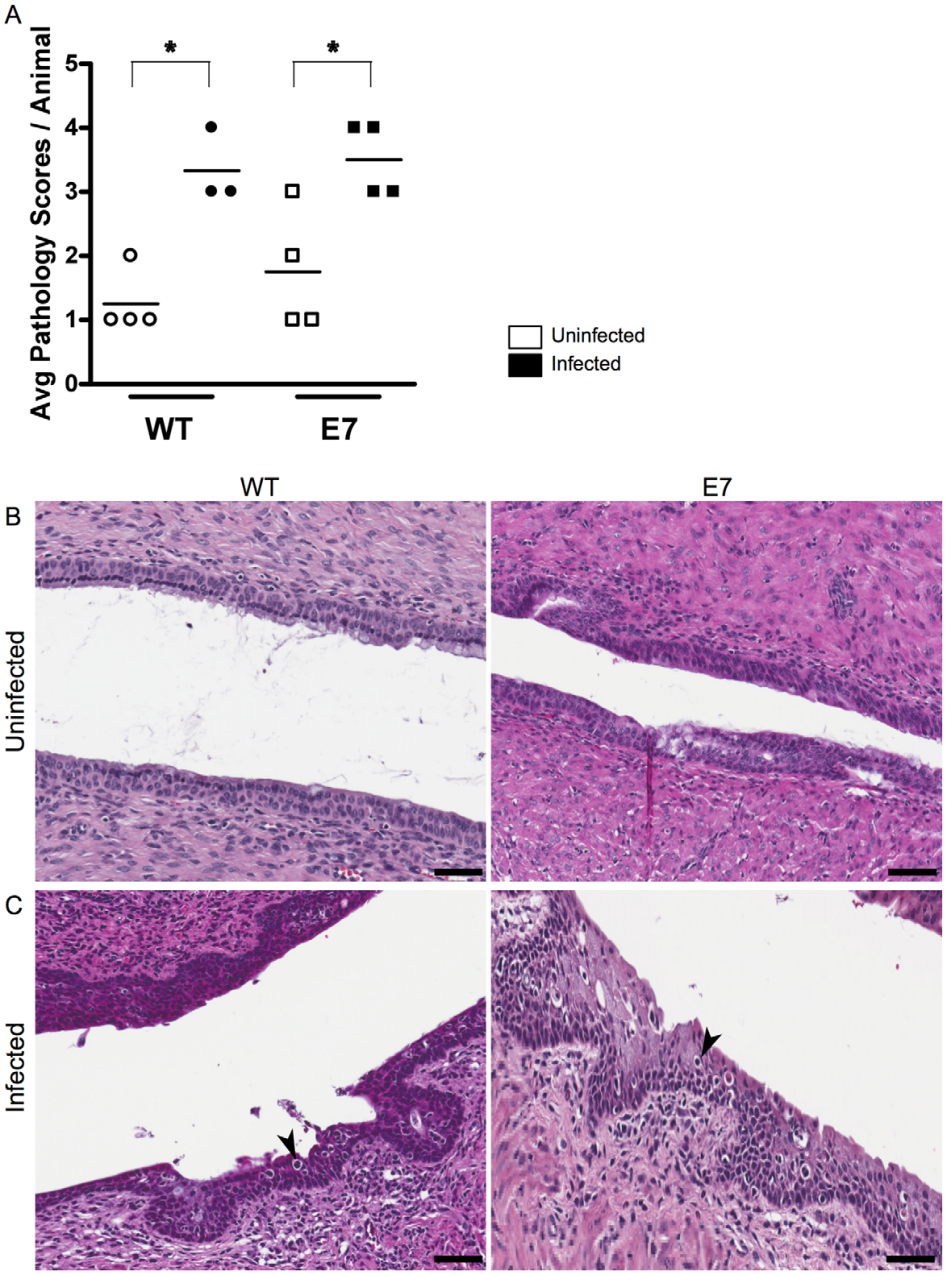
Presence of *Chlamydia muridarum* induces CIN. Infected and mock-infected groups of 3–4 K14-HPV-E7 mice and their wild-type littermates were sacrificed 7 days post-infection. H&E sections were evaluated by our pathologist (LJF) and each animal was given a score based on progression of cervical dysplasia; the scores were averaged for each animal. (A). The wild-type, mock-infected group received an average score of 1.3±0.3, N = 4, indicating these animals retained normal cervical epithelium after treatment. The wild-type infected group received an average score of 3.3±0.3, N = 3 representing a progression to CIN II, p = 0.0037. The K14-HPV-E7 mice followed a similar pattern with the uninfected group receiving a score of 1.8±0.5 indicating most of the animals had normal tissue, while the infected group received a score of 3.5±0.3 indicating these animals also progressed to CIN II, p = 0.0203, N = 4. (B) Uninfected tissue from a wild-type and K14-HPV-E7 mouse. (C) Infected tissue from the mice corresponds with epithelial projections into the stroma and contains cells that have increased nuclear:cytoplasm ratio (arrows). Scale bars, 50 μm.

We next investigated the effects of chlamydial infection on cervical histopathology. Cervical cancer arises from noninvasive premalignant lesions known as cervical intraepithelial neoplasias (CINs). In women these lesions are graded histologically based on the presence of atypical epithelial cells on the outer cervix: CIN I correlates with mild dysplasia, CIN II with moderate dysplasia, and CIN III corresponds to both severe dysplasia and carcinoma *in situ* [51]. A board certified pathologist blinded to experimental condition surveyed tissue from mock-infected and infected K14-HPV-E7 mice and their wild-type littermates, and assigned a pathological score to two separate sections from each animal. The rubric is detailed in the methods section. Scores were determined by a grading system developed specifically for HPV transgenic mice to determine the degree of dysplasia in the mouse cervix [52]. This classification system is based on the human model for carcinogenic progression mentioned above. We compared the scores between uninfected and infected animals to determine if chlamydial infection induced cervical dysplasia (Figure 4A). The wild-type, mock-infected group retained normal cervical epithelium after treatment, receiving an average score of 1.3±0.3. The wild-type infected group however, progressed to moderate cervical intraepithelial neoplasia with a CIN score of II receiving an average score of 3.3±0.3. The K14-HPV-E7 mice followed the same trend with the uninfected group receiving a score of 1.8±0.5 indicating the normal epithelium was only modestly affected by the transgene while the infected group received a score of 3.5±0.3 revealing these animals also progressed to CIN II. The normal tissue from both wild-type and transgenic animals contains cells with normal nuclear to cytoplasm ratio and mitotic figures present only in basal layers (Figure 4B). CIN II lesions contain cells with increased nuclear size, some anaplastic cells, and dysplastic cells distributed frequently throughout the squamous epithelium. The lesions are also characterized by epithelial projections thrown into the underlying cervical stroma (Figure 4C). The presence of moderate cervical dysplasia in the infected animals suggests that, at least initially, *Chlamydia* is able to directly cause cervical dysplasia progression. The slightly higher CIN II score the infected K14-HPV-E7 animals received also indicates that chlamydial infection could play a role in exacerbation of cellular defects contributed by HPV oncogene expression.

### Indication of Chlamydial Induced Cellular Defects *in Vivo*

**Figure 5:**
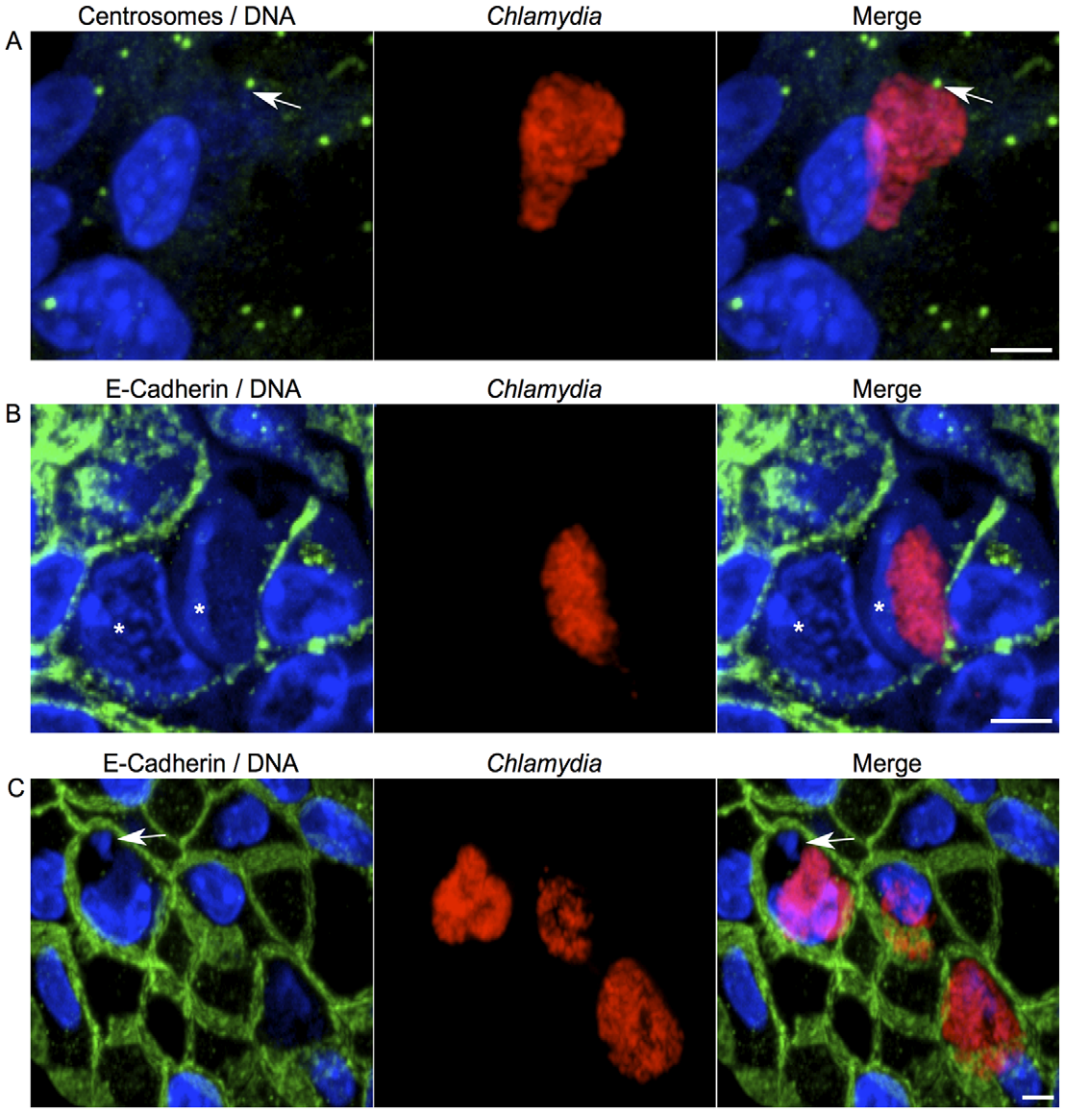
Evidence of centrosome mislocalization and genetic instability in infected animals. K14-HPV-E7 mice their wild-type littermates were infected on day 0 and reinfected on day 3, and the animals were examined for phenotypic evidence of precancerous characteristics within the cervix. (A) A 10 μm thick tissue section from a wild-type mouse was stained for centrosomes (green), *Chlamydia* (red), and DNA (blue). The arrow indicates the centrosome is localized to the chlamydial inclusion. (B) A K14-HPV-E7 tissue section was stained for E-Cadherin (green), *Chlamydia* (red), and DNA (blue). The stars denote two nuclei within the infected cell. The images in panel C are taken from wild-type tissue sections stained for E-cadherin (green), *Chlamydia* (red), and DNA (blue). The arrow indicates the formation of a micronuclei. Scale bars, 5 μm.

Finally, we wanted to determine if any of the phenotypic evidence of pre-cancerous and cancerous lesions we identified in cell culture existed *in vivo*. Because we showed *Chlamydia* was able to infect actively replicating cells and there was such an abundance of cell proliferation induced after infection we chose to infect the animals on day 0 and reinfect on day 3 of the one week infection. This increased the probability of infecting replicating cells. We then examined the vaginal and cervical tissue for the cellular defects that contribute to chromosomal instability. We infected both wild type and K14-HPV-E7 mice for these experiments. We evaluated 10 μm thick sections to allow us to visualize a layer of cells in its entirety. This allowed us to examine entire cells in three-dimensional space. Fluorescent confocal microscopy revealed evidence of centrosome mislocalization as we observed centrosomes associated with the chlamydial inclusion, rather than the juxtanuclear position they normally occupy (Figure 5A). This observation is consistent with an earlier observation of infected cells in culture where we previously reported that chlamydial infection leads to the physical separation of centrosomes, resulting in difficulty positioning them appropriately for cell division [23], [24]. We also observed infected cells with more than one nucleus (Figure 5B). Multinucleation is a phenotype associated with chromosomal instability, low and high grade cervical dysplasia, and we and others have shown chlamydial infection can induce multinucleation in cultured cells [25], [52], [53]. To establish that nuclei were, in fact, inside a single cell, we co-stained our sections with an antibody for the transmembrane protein E-cadherin. E-cadherin is expressed specifically in epithelial tissues and the antibody staining allowed us to visualize the membrane for individual cells. Further inspection of our tissue sections also resulted in the discovery of a micronucleus in an infected cell (Figure 5C). Micronuclei are small cytoplasmic bodies containing chromatin that are morphologically similar to nuclei, but are not included in daughter nuclei after cell division. They can result from mis-segregation of whole chromosomes during anaphase either due to a disruption in mitotic spindle assembly, defects in the spindle assembly checkpoint (SAC), or abnormal centrosome amplification [54]–[57]. Previous work to come out of our lab has demonstrated *Chlamydia* to be responsible for inducing spindle architecture defects, delaying the SAC, and stimulating centrosome amplification [24], [25]. We have also shown HeLa cells cured of an infection have increased rates of micronuclei formation [23]. The observation that many of these phenotypes occur during an *in vivo* infection of the mouse genital tract suggests these effects may be a contributing mechanism to cervical cancer.

## Discussion

The link between *Chlamydia trachomatis* and cervical cancer has been examined in several case-controlled and population based studies over the past decade and infection has been consistently associated with invasive cervical cancer (ICC), however a mechanism has yet to be established [12]–[19]. Chlamydial infections cause significant inflammation during infection, as well as permanent cytological changes in cultured cells. Both of these pathogenic effects have been linked to cancers in other systems.

Cervical cancer is intimately linked to infection with high risk HPV types such as 16 and 18. Over 90% of squamous cell carcinomas or adenocarcinomas of the cervix are positive for integrated high risk HPV genomes 16 and 18 [22]. The HPV encoded oncoproteins E6 and E7 are consistently expressed in these cancers and a significant role in transformation to malignancy is attributed to these proteins [53]. However, HPV infection alone is not sufficient to cause cervical cancer and cofactors such as other STD infections are epidemiologically linked to increased cancer rates [19], [22], [29], [58].

Chlamydial infection causes a number of cytological effects during infection of cells in culture. Our past studies have demonstrated that infection of both HeLa cells as well as normal human fibroblasts result in centrosome number defects [23]. The induction of multinucleation in chlamydial infected cells has been documented in both HeLa and mouse McCoy cells [34], [59]. In this study we show that these detrimental cellular defects, proliferation of centrosomes, formation of multipolar spindles, and the presence of multinucleated cells occur in all cultured cells tested and are independent of the expression of HPV E6, E7 or any other specific viral oncogene. Instead, the data presented here support the hypothesis that these cytological effects require only infection of a dividing cell population.

The cytological defects induced by infection, centrosome amplification, induction of multipolar spindles and multinucleation have all been linked to cellular transformation. The induction of anchorage independent growth in the 3T3 cellular transformation model to a level similar to UV radiation exposure suggests chlamydial infection acts as a potent mutagen, and as infection with *Coxiella burnetii* did not induce anchorage independent growth, suggests that this effect is specific to chlamydial infection. It is likely that the cytological changes that lead to chromosome instability are contributing factors in induction of transformation.

The epithelial lining of the genital tract is composed of terminally differentiated cells that undergo cyclical tissue remodeling leading to monthly cell turnover [45]. Because the epithelial cells are cyclically replaced by stem cells residing below the epithelial cell layer, *Chlamydia* was thought to have little access to dividing cells. Surprisingly, we found that although the number of replicating cells in the cervical transformation zone was low, we were able to identify a number of replicating infected cells, indicating that there is no barrier to the infection of the replicating cell population in this region of the mouse reproductive tract.

We hypothesize the replicating cell population in the cervical transformation zone would be the population likely to give rise to transformed cells after chlamydial infection. By using the E7 transgenic mice we increased the number of these target cells as these mice have hyperproliferative epithelial cells. We were surprised to see that chlamydial infection alone could induce hyperproliferation; this is likely the result of extensive tissue remodeling during and after the destructive events of infection. We were also a little surprised to find that chlamydial infection was able to cause moderate cervical intraepithelial neoplasia II in both the transgenic and wild-type mice. To the trained eye, hyperproliferation is easily distinguishable from the cytological changes induced during CIN II progression. We expected the K14-HPV-E7 mice to have some level of dysplasia due to the expression of the E7 oncogene, as well as the wild-type mice to experience some mild cell changes as a result of the chlamydial infection, but we did not expect both wild-type and transgenic infected mice to develop CIN II. We only assessed the mice at a one week time point, in future studies we will be interested to find out if this dysplasia persists or perhaps develops into carcinoma *in situ* over a 6 to 9 month period.

As the increased levels of cell replication upon infection create an environment predisposed for chlamydial induced centrosome aberrations, multipolar mitoses, and genetic instability, we were curious to find out whether chlamydial infection could induce the same phenotypic defects *in vivo* as we have reported *in vitro* [23]–[25]. By re-infecting both E7 transgenic and wild type mice after three days of infection we aimed to increase the number of replicating infected cells to increase the chance of observing these cytological defects *in vivo*. Indeed, we observed examples of multinucleation, micronuclei formation, as well as centrosome re-localization in the reinfected mice, suggesting the cytological phenotypes that lead to chromosome instability that we and others have documented are detectable in an infection model. However, even in these reinfected mice the number of infected dividing cells was low so we were unable to quantitate the frequency in which these events occurred.

We did not find any infected cells undergoing mitosis in our tissue sections so we were not able to directly observe spindle pole defects. This is likely because mitosis is a comparatively quick process and unlike cells in culture, where *Chlamydia* infects almost 100% of the cells, the level of infection is a great deal lower in animal tissue. Multinucleation, centrosome positioning defects, and the formation of micronuclei, however, are phenotypes that accumulate and are therefore more readily observed in the infected tissues. The discovery of multinucleated cells and micronuclei strongly suggest that chlamydial infection induces chromosomal instability and centrosome defects in tissue, just as it does in cultured cells.

We believe the combination of cellular proliferation induced by infection, and chlamydial induced centrosome and mitotic spindle defects promoting genomic instability, are likely contributing factors to cervical dysplasia. Furthermore, the *in vivo* evidence of chlamydial induced centrosome localization defects and genomic instability, which are present in precancerous and cancerous lesions of many origins, lends itself to the hypothesis that chlamydial infection may prime the cervix for progression to neoplasia or exacerbate neoplastic lesions already present. Whether these effects are sufficient to lead to invasive cervical cancer or whether progression is dependent on the co-expression of HPV oncogenes is a question to address in future long term experiments. Additionally, the mechanism of clearance of the infection whether naturally or with antibiotic treatment could be a contributing factor in the accumulation of cells with chlamydial induced cytological defects.

## Materials and Methods

### Organisms and Cell Culture

*Chlamydia trachomatis* serovar L2 (LGV 434), and *C. muridarum* Nigg strain (referred to as MoPn or mouse pneumonitis) were grown in McCoy cells and elementary bodies (EBs) were purified by Renografin density gradient centrifugation as previously described [60]. EBs were stored at −80 C until ready for use. *Coxiella burnetii* Nine Mile phase II (NMII) clone 4 was a gift from Robert Heinzen, Rocky Mountain Labs, NIAID/NIH.

All cell lines were obtained from American Type Culture Collection. McCoy cells were maintained in DMEM (Gibco), supplemented with 10% FBS (Cellgro) and 10 μg/mL gentamicin (Cellgro). 3T3 (CCL-92) and COS-7 (CRL-1651) cells were maintained in RPMI-1640 meduim (Cellgro), supplemented with 10% FBS and 10 μg/mL gentamicin. End1/E6E7 (CRL-2615) cells were maintained in serum-free Keratinocyte Medium (ScienCell) supplemented with Keratinocyte Growth Supplement (ScienCell) and 10 μg/mL gentamicin.

### Infection of Cultured Cells

Confluent monolayers of cells were incubated with *C. trachomatis* EBs at an MOI of ∼5 in Hank's Balanced Salt Solution (Gibco) for 30 minutes at room temperature while rocking. After incubation, the HBSS was removed and replaced with fresh complete media, and the infection was allowed to continue for 36 hours. *Coxiella burnetii* infections were carried out similarly, however cells were incubated with the inoculum for 4 hours and then replaced with fresh media containing no antibiotics. *C. burnetii* infections were allowed to continue for 96 hours.

### Immunofluorescence Staining of Cultured Cells

Cells for fluorescent microscopy were grown on 12-mm number 1.5 borosilicate glass coverslips coated with Poly-L-lysine (Sigma). The coverslips were fixed in ice cold methanol for 10 minutes, and incubated with the primary antibodies as follows: mouse monoclonal anti-γ-tubulin (Sigma), mouse monoclonal anti-β-tubulin (Sigma). *Chlamydia trachomatis* was stained with human serum from male AB plasma purchased from Sigma. To visualize the primary antibodies appropriate AlexaFluor (Molecular Probes/Life Technologies) conjugated secondary antibodies were used; 488/568/647 against mouse, or human immunoglobulin G (IgG). The far-red fluorescent DNA dye DRAQ5 (Biostatus Limited) was used to visualize nuclei.

### Microscope

Images were acquired using a spinning disk confocal system connected to a Leica DMIRB microscope with a 63x oil-immersion objective, equipped with a Photometrics cascade-cooled EMCCD camera, under the control of the Open Source software package μManager (http://www.micro-manager.org/). Images were processed using the image analysis software ImageJ (http://rsb.info.nih.gov/ij/). Representative confocal micrographs displayed in the figures are maximal intensity projections of the 3D data sets, unless otherwise noted.

### Soft Agar Assay

The 3T3 soft agar transformation assay (Millipore) was performed according the manufacturer's instructions. Briefly, 3T3 cells were mock-infected, infected (*C. trachomatis* or *C. burnetii*), or treated with UV for 1, 3, and 5 minutes. The *C. trachomatis*, and *C. burnetii* infections were cured for 3–4 days with 50 μg/mL and 10 μg/mL rifampicin (Fisher Scientific), respectively. The cells were allowed to recover from antibiotic treatment for 2–3 days and then plated with the appropriate controls onto a minimum of four 6-well plates containing soft agar. The soft agar plates were fed with fresh media every 3–4 days, and incubated for approximately 28 days. After the incubation the cells were stained with a commercially available kit (Millipore) and the number of colonies per well were counted. These assays were repeated on 3–6 independent occasions. The efficacy of the antibiotic treatment was verified by microscopy and a replating assay. Infected and cured 3T3 cells were co-stained with human serum and the DNA dye DRAQ5 to verify no chlamydial inclusions remained after rifampicin treatment. Infected and cured 3T3 cells were sonicated and replated onto uninfected 3T3 cells to determine the infectivity of the cured cells.

### Mice

K14-HPV-E7 mice were a obtained from Paul Lambert, McArdle Laboratory for Cancer Research, University of Wisconsin Medical School, Madison, Wisconsin, and were generated as described previously [49]. The transgene was maintained in a hemizygous state on the inbred FVB/N background. All mice were housed in American Association for Accreditation of Laboratory Animal Care-approved facilities at the University of Florida, and all animal manipulations were carried out in accordance with the Institutional Animal Care and Use Committee approved protocol 20081415. University of Florida Animal Care Services provided assistance with implantation of estrogen pellets.

### Infection and Experimental Manipulation of Mice

Groups of 8–10 week old female K14-HPV-E7 mice and their wild-type littermates were injected intraperitoneally with 2.5 mg Depo-Provera (Pfizer) in 100 μL sterile phosphate buffered saline (PBS) at 10, and 3 days prior to infection. At 6 days prior to infection, mice were anesthetized with isoflurane and continuous release estrogen pellets delivering 0.05 mg 17ß-estradiol over 60 days (Innovative Research of America) were implanted subcutaneously in the dorsal skin. The mice were then infected via the vaginal vault with 1×10^5^ inclusion-forming units (IFU) of *Chlamydia muridarum* (MoPn). Control groups for each set of mice were mock-infected with sucrose phosphate glutamate buffer (SPG) 8 mM sodium phosphate dibasic anhydrous, 2 mM sodium phosphate monobasic, 220 mM sucrose, 0.5 mM L-glutamic acid]. Mice were sacrificed at 1 week post-infection. For three consecutive days prior to sacrifice, all groups received intraperitoneal injections of 200 μg EdU (5-ethynyl-2′-deoxyuridine, Life Technologies C10337) in 50 μL sterile PBS.

The mice represented in Figure 5 received no estrogen treatment and were sacrificed 5 days after initial infection. Infection was carried out as above, however infected groups were boosted on day 3 with an additional 1×10^5^ IFU of *Chlamydia muridarum*.

### Verification of Infection

Verification of genital tract infection was adapted from the Morrison laboratory [61], and monitored as follows. The vaginal canal was swabbed with a calcium alginate tipped swab (Fisher Scientific), the tip was then vortexed in 500 μL SPG with two sterile 4 mm glass beads (Kimble Chase) for 2 minutes. Each sample was then diluted appropriately and 300 μLof inoculum was placed onto McCoy cells. The plates were centrifuged at 900×g for 1 hour, followed by incubation at 37 C for 1 hour. After incubation the inoculum was removed and 500 μLof fresh media [DMEM supplemented with 10% FBS, 15 μg/mL gentamicin, and 0.25 μg/mL Fungizone (amphotericin B, Life Technologies)] was added. The infections were allowed to continue for 30 hours. The cultures were fixed with methanol and the number of IFUs was determined by indirect immunofluorescence as described above.

### Histopathology

Whole murine reproductive tracts were harvested following sacrifice and placed in embedding cassettes (Fisher Scientific) with one sponge for compression. Reproductive tracts were fixed overnight at room temperature in 10% neutral buffered formalin (Fisher Scientific). The University of Florida Molecular Pathology Core performed paraffin-embedding, sectioning, and hematoxylin and eosin (H&E) staining of all tissues. Paraffin blocks were sectioned, with the entire organ in one plane, at 4 and 10 μm as specified in the figure legends. Two H&E slides, sectioned at different depths, from each animal were evaluated by a board certified pathologist (LJF) blinded to experimental condition. Cervical intraepithelial neoplasia (CIN) scores were determined according to the published system developed with K14-HPV-E7 mice [52]. The grading system ranged from 1–6 arbitrary units and assessed the nucleus:cytoplasm ratio within squamous epithelial cells, the frequency of these cells in the squamous epithelium, and the architecture of the intersection between squamous epithelium and underlying vaginal or cervical stroma. A score of 1 corresponded to normal tissue, 2 =  CIN I, 3 =  CIN II, 4 =  CIN III, score of 5 represented carcinoma *in situ* (CIS), and squamous cell carcinoma (SCC) received a score of 6.

### Immunofluorescence Staining of Mouse Reproductive Tracts

Formalin-fixed, paraffin-embedded tissue sections were deparaffinized in two changes of xylene for 10 minutes each and then dehydrated in 100% ethanol for 5 minutes. The slides were then rehydrated through a graded ethanol series (95% and 70%) for 5 minutes each, followed by a wash in dH20 for 5 minutes; all steps were carried out at room temperature. The slides were placed in sodium citrate buffer [10 mM sodium citrate, 0.05% Tween 20, pH 6.0] at 96.5 C for 25 minutes. To allow the slides to cool to room temperature they were moved to a dH20 bath for 5 minutes. Sections were permeabilized overnight at 4 C with 0.1% Triton X-100 (Fisher Scientific) in 1X PBS. The slides were washed in 0.5% PBS-Tween 20 (Fisher Scientific) (PBST), and then blocked with 10% normal goat serum (Life Technologies) at room temperature for a minimum of 4 hours. Slides were washed with PBST and incubated with primary antibodies diluted in 10% normal goat serum for 24 hours. Primary antibodies consisted of mouse monoclonal anti-γ-tubulin (Sigma), and mouse monoclonal anti-E-Cadherin (BD Biosciences). *Chlamydia trachomatis* was stained with human serum from male AB plasma purchased from Sigma. Tissues were washed three times for 30 minutes each in PBST and incubated in secondary antibody for 4–8 hours. To visualize the primary antibodies appropriate AlexaFluor (Molecular Probes/Life Technologies) conjugated secondary antibodies were used; 488/568/647 against mouse, or human immunoglobulin G (IgG). The far-red fluorescent DNA dye DRAQ5 (Biostatus Limited) was used to visualize nuclei.

### Calculation of Cell Proliferation

The rate of cell proliferation in mice was determined by uptake of the thymidine analog EdU (5-ethynyl-2′-deoxyuridine), with the use of the Click-iT EdU kit per manufacturer's instructions (Life Technologies). Briefly, tissue sections were treated for immunofluorescence as above, however before incubation with primary antibody, the Click-iT reaction was performed to visualize EdU positive cells. For each animal, EdU positive cells were compared with the total number of cells present in a field of view. A minimum of 2000 cells were counted over 15–20 fields, and this was completed at two different depths within the transformation zone.

### Statistical Analyses

Numerical data are presented as the mean ± SEM, unless otherwise noted, and were analyzed by the unpaired Student's t-test to compare means between two groups using GraphPad Prism4 software, version 4.03 for Windows (GraphPad Software, San Diego, CA).

## Supporting Information

Figure S1 **Rifampicin treated 3T3 cells are effectively cured of chlamydial infection.** (A) 3T3 cells infected with *Chlamydia trachomatis* for 36 hours were stained for microtubules (green), *Chlamydia* (red), and DNA (blue). (B) Infected cells were cured with 50 μg/mL rifampicin for 4 days, and then co-stained for microtubules (green), *Chlamydia* (red), and DNA (blue). Scale bars, 5 μm.
(TIFF)

## References

[1] Bébéar C, de Barbeyrac B (2009) Genital Chlamydia trachomatis infections. Clin Microbiol Infect 15: 4–10 doi:10.1111/j.1469-0691.2008.02647.x. 10.1111/j.1469-0691.2008.02647.x 19220334

[2] Ohman H, Tiitinen A, Halttunen M, Lehtinen M, Paavonen J, et al (2009) Cytokine polymorphisms and severity of tubal damage in women with Chlamydia-associated infertility. J Infect Dis 199: 1353–1359 doi:10.1086/597620. 19358670 10.1086/597620

[3] Miller WC, Ford CA, Morris M, Handcock MS, Schmitz JL, et al (2004) Prevalence of chlamydial and gonococcal infections among young adults in the United States. JAMA 291: 2229–2236 doi:10.1001/jama.291.18.2229. 15138245 10.1001/jama.291.18.2229

[4] Datta SD, Torrone E, Kruszon-Moran D, Berman S, Johnson R, et al (2012) Chlamydia trachomatis trends in the United States among persons 14 to 39 years of age, 1999–2008. Sex Transm Dis 39: 92–96 doi:10.1097/OLQ.0b013e31823e2ff7. 22249296 10.1097/OLQ.0b013e31823e2ff7

[5] World Health Organization Department of HIV/AIDS © 2001 (2001) Global Prevalence and Incidence of Selected Curable Sexually Transmitted Infections: Overview and Estimates: 1–50.

[6] Patton DL, Landers DV, Schachter J (1989) Experimental Chlamydia trachomatis salpingitis in mice: initial studies on the characterization of the leukocyte response to chlamydial infection. J Infect Dis 159: 1105–1110. 2656878 10.1093/infdis/159.6.1105

[7] Cotter TW, Meng Q, Shen ZL, Zhang YX, Su H, et al (1995) Protective efficacy of major outer membrane protein-specific immunoglobulin A (IgA) and IgG monoclonal antibodies in a murine model of Chlamydia trachomatis genital tract infection. Infect Immun 63: 4704–4714. 7591126 10.1128/iai.63.12.4704-4714.1995 PMC173675

[8] Shah AA, Schripsema JH, Imtiaz MT, Sigar IM, Kasimos J, et al (2005) Histopathologic changes related to fibrotic oviduct occlusion after genital tract infection of mice with Chlamydia muridarum. Sex Transm Dis 32: 49–56. 15614121 10.1097/01.olq.0000148299.14513.11

[9] Stephens RS, Kalman S, Lammel C, Fan J, Marathe R, et al (1998) Genome sequence of an obligate intracellular pathogen of humans: Chlamydia trachomatis. Science 282: 754–759. 9784136 10.1126/science.282.5389.754

[10] Collingro A, Tischler P, Weinmaier T, Penz T (2011) Unity in Variety – the Pan-Genome of the Chlamydiae. Molecular biology and Evolution 28: 12 doi: 10.1093/molbev/msr161. 10.1093/molbev/msr161 PMC3247790 21690563

[11] Read TD, Brunham RC, Shen C, Gill SR, Heidelberg JF, et al (2000) Genome sequences of Chlamydia trachomatis MoPn and Chlamydia pneumoniae AR39. Nucleic Acids Res 28: 1397–1406. 10684935 10.1093/nar/28.6.1397 PMC111046

[12] Koskela P, Anttila T, Bjørge T, Brunsvig A, Dillner J, et al (2000) Chlamydia trachomatis infection as a risk factor for invasive cervical cancer. Int J Cancer 85: 35–39. 10585579 10.1002/(sici)1097-0215(20000101)85:1<35::aid-ijc6>3.0.co;2-a

[13] Anttila T, Saikku P, Koskela P, Bloigu A, Dillner J, et al (2001) Serotypes of Chlamydia trachomatis and risk for development of cervical squamous cell carcinoma. JAMA 285: 47–51. 11150108 10.1001/jama.285.1.47

[14] Smith JS, Bosetti C, Muñoz N, Herrero R, Bosch FX, et al (2004) Chlamydia trachomatis and invasive cervical cancer: a pooled analysis of the IARC multicentric case-control study. Int J Cancer 111: 431–439 doi:10.1002/ijc.20257. 15221973 10.1002/ijc.20257

[15] Smith JS, Muñoz N, Herrero R, Eluf-Neto J, Ngelangel C, et al (2002) Evidence for Chlamydia trachomatis as a human papillomavirus cofactor in the etiology of invasive cervical cancer in Brazil and the Philippines. J Infect Dis 185: 324–331 doi:10.1086/338569. 11807714 10.1086/338569

[16] Wallin KL, Wiklund F, Luostarinen T, Angström T, Anttila T, et al (2002) A population-based prospective study of Chlamydia trachomatis infection and cervical carcinoma. Int J Cancer 101: 371–374 doi:10.1002/ijc.10639. 12209962 10.1002/ijc.10639

[17] Matsumoto K, Yasugi T, Oki A, Hoshiai H, Taketani Y, et al (2003) Are smoking and chlamydial infection risk factors for CIN? Different results after adjustment for HPV DNA and antibodies. Br J Cancer 89: 831–833 doi:10.1038/sj.bjc.6601220. 12942113 10.1038/sj.bjc.6601220 PMC2394484

[18] Hinkula M, Pukkala E, Kyyrönen P, Laukkanen P, Koskela P, et al (2004) A population-based study on the risk of cervical cancer and cervical intraepithelial neoplasia among grand multiparous women in Finland. Br J Cancer 90: 1025–1029 doi:10.1038/sj.bjc.6601650. 14997202 10.1038/sj.bjc.6601650 PMC2410219

[19] Madeleine MM, Anttila T, Schwartz SM, Saikku P, Leinonen M, et al (2007) Risk of cervical cancer associated with Chlamydia trachomatis antibodies by histology, HPV type and HPV cofactors. Int J Cancer 120: 650–655 doi:10.1002/ijc.22325. 17096345 10.1002/ijc.22325 PMC4049152

[20] Woodman CBJ, Collins SI, Young LS (2007) The natural history of cervical HPV infection: unresolved issues. Nat Rev Cancer 7: 11–22 doi:10.1038/nrc2050. 17186016 10.1038/nrc2050

[21] Walboomers JM, Jacobs MV, Manos MM, Bosch FX, Kummer JA, et al.. (1999) AID-PATH431>3.0.CO;2-F.

[22] Hausen zur H (1996) Papillomavirus infections–a major cause of human cancers. Biochim Biophys Acta 1288: F55–F78. 8876633 10.1016/0304-419x(96)00020-0

[23] Grieshaber SS, Grieshaber NA, Miller N, Hackstadt T (2006) Chlamydia trachomatis causes centrosomal defects resulting in chromosomal segregation abnormalities. Traffic 7: 940–949 doi:10.1111/j.1600-0854.2006.00439.x. 16882039 10.1111/j.1600-0854.2006.00439.x

[24] Knowlton AE, Brown HM, Richards TS, Andreolas LA, Patel RK, et al (2011) Chlamydia trachomatis infection causes mitotic spindle pole defects independently from its effects on centrosome amplification. Traffic 12: 854–866 doi:10.1111/j.1600-0854.2011.01204.x. 21477082 10.1111/j.1600-0854.2011.01204.x PMC3116664

[25] Brown HM, Knowlton AE, Grieshaber SS (2012) Chlamydial Infection Induces Host Cytokinesis Failure at Abscission. Cell Microbiol. doi:10.1111/j.1462-5822.2012.01820.x. 10.1111/j.1462-5822.2012.01820.x PMC3443326 22646503

[26] Pihan GA, Purohit A, Wallace J, Knecht H, Woda B, et al (1998) Centrosome defects and genetic instability in malignant tumors. Cancer Res 58: 3974–3985. 9731511

[27] Pihan G, Doxsey SJ (2003) Mutations and aneuploidy: co-conspirators in cancer? Cancer Cell 4: 89–94. 12957283 10.1016/s1535-6108(03)00195-8

[28] Lingle WL, Barrett SL, Negron VC, D'Assoro AB, Boeneman K, et al (2002) Centrosome amplification drives chromosomal instability in breast tumor development. Proc Natl Acad Sci USA 99: 1978–1983 doi:10.1073/pnas.032479999. 11830638 10.1073/pnas.032479999 PMC122305

[29] Hausen zur H (2002) Papillomaviruses and cancer: from basic studies to clinical application. Nat Rev Cancer 2: 342–350 doi:10.1038/nrc798. 12044010 10.1038/nrc798

[30] Nigg EA (2006) Origins and consequences of centrosome aberrations in human cancers. Int J Cancer 119: 2717–2723 doi:10.1002/ijc.22245. 17016823 10.1002/ijc.22245

[31] Ganem NJ, Godinho SA, Pellman D (2009) A mechanism linking extra centrosomes to chromosomal instability. Nature 460: 278–282 doi:10.1038/nature08136. 19506557 10.1038/nature08136 PMC2743290

[32] Schwarz E, Freese UK, Gissmann L, Mayer W, Roggenbuck B, et al (1985) Structure and transcription of human papillomavirus sequences in cervical carcinoma cells. Nature 314: 111–114. 2983228 10.1038/314111a0

[33] Duensing S, Münger K (2002) Human papillomaviruses and centrosome duplication errors: modeling the origins of genomic instability. Oncogene 21: 6241–6248 doi:10.1038/sj.onc.1205709. 12214255 10.1038/sj.onc.1205709

[34] Greene W, Zhong G (2003) Inhibition of host cell cytokinesis by Chlamydia trachomatis infection. J Infect 47: 45–51. 12850162 10.1016/s0163-4453(03)00039-2

[35] Johnson KA, Tan M, Sütterlin C (2009) Centrosome abnormalities during a Chlamydia trachomatis infection are caused by dysregulation of the normal duplication pathway. Cell Microbiol 11: 1064–1073 doi:10.1111/j.1462-5822.2009.01307.x. 19290915 10.1111/j.1462-5822.2009.01307.x PMC3308718

[36] Fichorova RN, Rheinwald JG, Anderson DJ (1997) Generation of papillomavirus-immortalized cell lines from normal human ectocervical, endocervical, and vaginal epithelium that maintain expression of tissue-specific differentiation proteins. Biol Reprod 57: 847–855. 9314589 10.1095/biolreprod57.4.847

[37] Gluzman Y (1981) SV40-transformed simian cells support the replication of early SV40 mutants. Cell 23: 175–182. 6260373 10.1016/0092-8674(81)90282-8

[38] Todaro GJ, Green H (1963) Quantitative studies of the growth of mouse embryo cells in culture and their development into established lines. J Cell Biol 17: 299–313. 13985244 10.1083/jcb.17.2.299 PMC2106200

[39] DiPaolo JA, Nelson RL, Donovan PJ (1969) Sarcoma-producing cell lines derived from clones transformed in vitro by benzo[a]pyrene. Science 165: 917–918. 5798317 10.1126/science.165.3896.917

[40] Lasne C, Gentil A, Chouroulinkov I (1974) Two-stage malignant transformation of rat fibroblasts in tissue culture. Nature 247: 490–491. 4856407 10.1038/247490a0

[41] Berwald Y, Sachs L (1963) In Vitro Cell Transformation With Chemical Carcinogens. Nature 200: 1182–1184. 14089903 10.1038/2001182a0

[42] Sasaki K, Bohnenberger S, Hayashi K, Kunkelmann T, Muramatsu D, et al (2012) Recommended protocol for the BALB/c 3T3 cell transformation assay. Mutat Res 744: 30–35 doi:10.1016/j.mrgentox.2011.12.014. 22212201 10.1016/j.mrgentox.2011.12.014

[43] Shin SI, Freedman VH, Risser R, Pollack R (1975) Tumorigenicity of virus-transformed cells in nude mice is correlated specifically with anchorage independent growth in vitro. Proc Natl Acad Sci USA 72: 4435–4439. 172908 10.1073/pnas.72.11.4435 PMC388736

[44] Heinzen RA, Hackstadt T, Samuel JE (1999) Developmental biology of Coxiella burnettii. Trends in Microbiology 7: 149–154. 10217829 10.1016/s0966-842x(99)01475-4

[45] Leppert PC (2012) Tissue remodeling in the female reproductive tract–a complex process becomes more complex: the role of Hox genes. Biol Reprod 86: 98 doi:10.1095/biolreprod.112.099283. 22302691 10.1095/biolreprod.112.099283

[46] Salic A, Mitchison TJ (2008) A chemical method for fast and sensitive detection of DNA synthesis in vivo. Proc Natl Acad Sci USA 105: 2415–2420 doi:10.1073/pnas.0712168105. 18272492 10.1073/pnas.0712168105 PMC2268151

[47] Autier P, Coibion M, Huet F, Grivegnee AR (1996) Transformation zone location and intraepithelial neoplasia of the cervix uteri. Br J Cancer 74: 488–490. 8695371 10.1038/bjc.1996.388 PMC2074626

[48] Elson DA, Riley RR, Lacey A, Thordarson G, Talamantes FJ, et al (2000) Sensitivity of the cervical transformation zone to estrogen-induced squamous carcinogenesis. Cancer Res 60: 1267–1275. 10728686

[49] Herber R, Liem A, Pitot H, Lambert PF (1996) Squamous epithelial hyperplasia and carcinoma in mice transgenic for the human papillomavirus type 16 E7 oncogene. The Journal of Virology 70: 1873–1881. 8627712 10.1128/jvi.70.3.1873-1881.1996 PMC190015

[50] Brake T, Lambert PF (2005) Estrogen contributes to the onset, persistence, and malignant progression of cervical cancer in a human papillomavirus-transgenic mouse model. Proc Natl Acad Sci USA 102: 2490–2495 doi:10.1073/pnas.0409883102. 15699322 10.1073/pnas.0409883102 PMC548999

[51] Steenbergen RDM, de Wilde J, Wilting SM, Brink AATP, Snijders PJF, et al (2005) HPV-mediated transformation of the anogenital tract. J Clin Virol 32 Suppl 1S25–S33 doi:10.1016/j.jcv.2004.11.019. 15753009 10.1016/j.jcv.2004.11.019

[52] Riley RR, Duensing S, Brake T, Münger K, Lambert PF, et al (2003) Dissection of human papillomavirus E6 and E7 function in transgenic mouse models of cervical carcinogenesis. Cancer Res 63: 4862–4871. 12941807

[53] Duensing S, Duensing A, Crum CP, Münger K (2001) Human papillomavirus type 16 E7 oncoprotein-induced abnormal centrosome synthesis is an early event in the evolving malignant phenotype. Cancer Res 61: 2356–2360. 11289095

[54] Kirsch-Volders M, Elhajouji A, Cundari E, Van Hummelen P (1997) The in vitro micronucleus test: a multi-endpoint assay to detect simultaneously mitotic delay, apoptosis, chromosome breakage, chromosome loss and non-disjunction. Mutat Res 392: 19–30. 9269328 10.1016/s0165-1218(97)00042-6

[55] Gisselsson D, Håkanson U, Stoller P, Marti D, Jin Y, et al (2008) When the genome plays dice: circumvention of the spindle assembly checkpoint and near-random chromosome segregation in multipolar cancer cell mitoses. PLoS ONE 3: e1871 doi:10.1371/journal.pone.0001871. 18392149 10.1371/journal.pone.0001871 PMC2289843

[56] Zyss D, Gergely F (2009) Centrosome function in cancer: guilty or innocent? Trends in Cell Biology 19: 334–346 doi:10.1016/j.tcb.2009.04.001. 19570677 10.1016/j.tcb.2009.04.001

[57] Fenech M, Bonassi S (2011) The effect of age, gender, diet and lifestyle on DNA damage measured using micronucleus frequency in human peripheral blood lymphocytes. Mutagenesis 26: 43–49 doi:10.1093/mutage/geq050. 21164181 10.1093/mutage/geq050

[58] Roden R, Wu TC (2006) How will HPV vaccines affect cervical cancer? Nat Rev Cancer 6: 753–763 doi:10.1038/nrc1973. 16990853 10.1038/nrc1973 PMC3181152

[59] Campbell S, Richmond SJ, Yates PS (1989) The effect of Chlamydia trachomatis infection on the host cell cytoskeleton and membrane compartments. J Gen Microbiol 135: 2379–2386. 2483409 10.1099/00221287-135-9-2379

[60] Howard L, Orenstein NS, King NW (1974) Purification on renografin density gradients of Chlamydia trachomatis grown in the yolk sac of eggs. Appl Microbiol 27: 102–106. 4855645 10.1128/am.27.1.102-106.1974 PMC379975

[61] Farris CM, Morrison RP (2011) Vaccination against Chlamydia genital infection utilizing the murine C. muridarum model. Infect Immun 79: 986–996 doi:10.1128/IAI.00881-10. 21078844 10.1128/IAI.00881-10 PMC3067520

