# Supplementary figures and images for: Chlamydia Induces Anchorage Independence in 3T3 Cells and Detrimental Cytological Defects in an Infection Model
Source: PLoS One. 2013 Jan 7;8(1):e54022. doi: 10.1371/journal.pone.0054022 (PMC3538680; doi:10.1371/journal.pone.0054022)

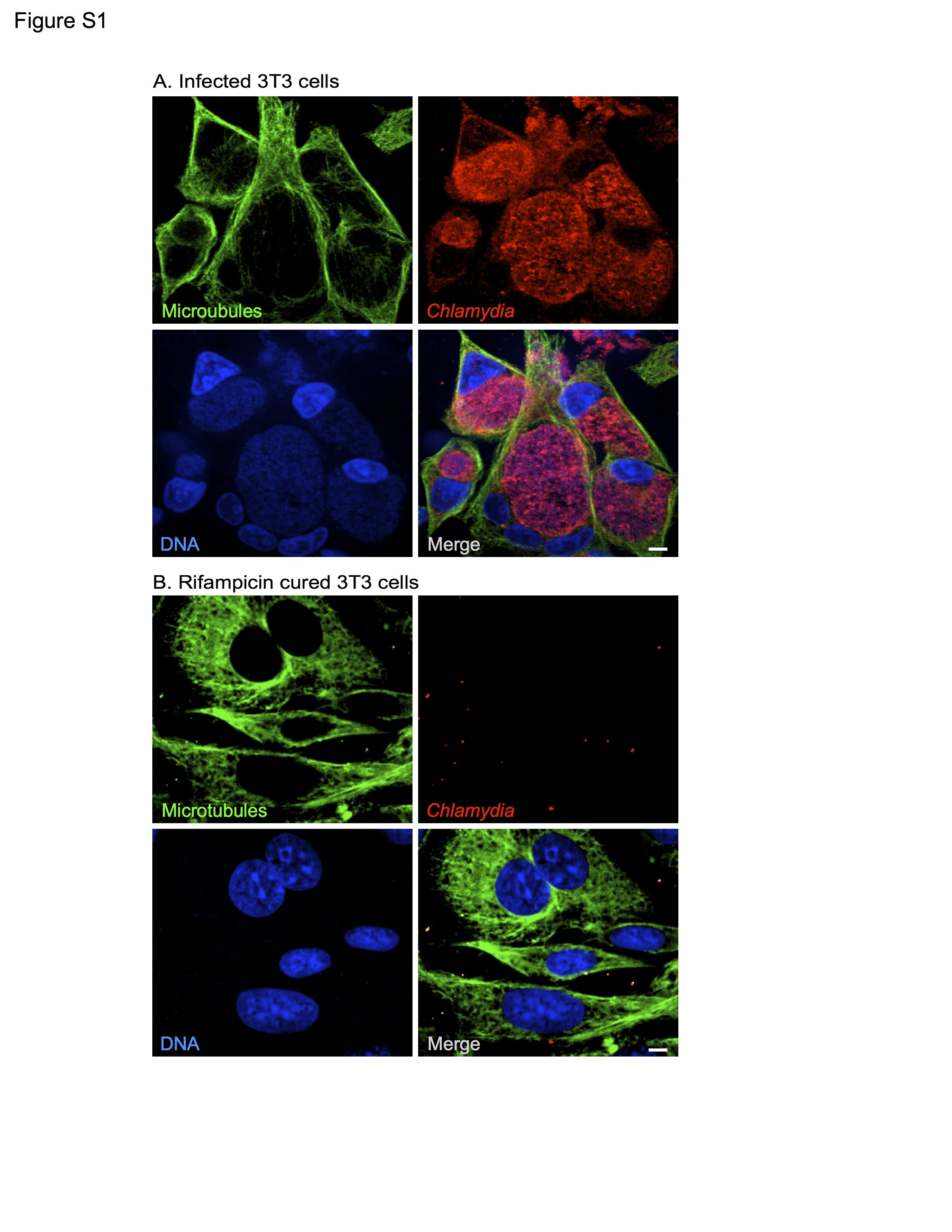

Supplement: Figure S1 — Rifampicin treated 3T3 cells are effectively cured of chlamydial infection. (A) 3T3 cells infected with Chlamydia trachomatis for 36 hours were stained for microtubules (green), Chlamydia (red), and DNA (blue). (B) Infected cells were cured with 50 μg/mL rifampicin for 4 days, and then co-stained for microtubules (green), Chlamydia (red), and DNA (blue). Scale bars, 5 μm. (TIFF) [file pone.0054022.s001.tiff]
